# Downfield Proton MRSI at 3 Tesla: A Pilot Study in Human Brain Tumors

**DOI:** 10.3390/cancers15174311

**Published:** 2023-08-29

**Authors:** İpek Özdemir, David O. Kamson, Semra Etyemez, Lindsay Blair, Doris D. M. Lin, Peter B. Barker

**Affiliations:** 1 Russell H. Morgan Department of Radiology and Radiological Science, The Johns Hopkins University School of Medicine, Baltimore, MD 21205, USA; 2 Sidney Kimmel Comprehensive Cancer Center, The Johns Hopkins University School of Medicine, Baltimore, MD 21205, USA; 3 Department of Obstetrics & Gynecology, Weill Cornell Medicine, New York, NY 10065, USA; 4 Department of Psychiatry, Weill Cornell Medicine, New York, NY 10065, USA; 5 F.M. Kirby Center for Functional Brain MRI, The Kennedy Krieger Institute, Baltimore, MD 21205, USA

**Keywords:** brain tumors, downfield magnetic resonance spectroscopic imaging, glioblastoma, tumor recurrence

## Abstract

**Simple Summary:**

This paper describes a new MR imaging technique known as downfield MR spectroscopic imaging (DF-MRSI) that has only recently been shown to be possible in the human brain on commonly available 3 Tesla MRI scanners. This is the first application of this methodology to human brain tumors.

**Abstract:**

Purpose: To investigate the use of 3D downfield proton magnetic resonance spectroscopic imaging (DF-MRSI) for evaluation of tumor recurrence in patients with glioblastoma (GBM). Methods: Seven patients (4F, age range 44–65 and mean ± standard deviation 59.3 ± 7.5 years) with previously treated GBM were scanned using a recently developed 3D DF-MRSI sequence at 3T. Short TE 3D DF-MRSI and water reference 3D-MRSI scans were collected with a nominal spatial resolution of 0.7 cm^3^. DF volume data in eight slices covered 12 cm of brain in the cranio-caudal axis. Data were analyzed using the ‘LCModel’ program and a basis set containing nine peaks ranging in frequency between 6.83 to 8.49 ppm. The DF8.18 (assigned to amides) and DF7.90 peaks were selected for the creation of metabolic images and statistical analysis. Longitudinal MR images and clinical history were used to classify brain lesions as either recurrent tumor or treatment effect, which may include necrosis. DF-MRSI data were compared between lesion groups (recurrent tumor, treatment effect) and normal-appearing brain. Results: Of the seven brain tumor patients, two were classified as having recurrent tumor and the rest were classified as treatment effect. Amide metabolite levels from recurrent tumor regions were significantly (*p* < 0.05) higher compared to both normal-appearing brain and treatment effect regions. Amide levels in lesion voxels classified as treatment effect were significantly lower than normal brain. Conclusions: 3D DF-MRSI in human brain tumors at 3T is feasible and was well tolerated by all patients enrolled in this preliminary study. Amide levels measured by 3D DF-MRSI were significantly different between treatment effect and tumor regrowth.

## 1. Introduction

Glioblastoma (GBM) is the most malignant and frequently encountered primary brain tumor in adults [1]. Maximal possible surgical resection, followed by radiation therapy (XRT) and temozolomide (TMZ) chemotherapy, is the standard of care treatment [2,3]. Despite intensive research effort, overall survival for patients with GBM remains at around 15 months [4,5,6]. Currently, assessment of therapy response in GBM is performed primarily using MRI. However, recurrent tumor and effects of treatment (including radiation necrosis) may have similar appearance on MRI and are often difficult to distinguish as a result. Interpretation of MRI is also complicated by the commonly encountered phenomena of ‘pseudoprogression’ and ‘pseudoresponse’ [7]. Therefore, accurate differentiation of recurrent tumor from treatment effects, including radiation necrosis and edema, remains challenging [8,9].

Due to this, other advanced MR techniques including diffusion-weighted MRI (DWI), MR perfusion imaging (PWI), conventional upfield (<4.7 ppm) magnetic resonance spectroscopy (MRS), and more recently magnetization transfer [10,11], amide-proton transfer chemical exchange saturation transfer (APT-CEST) MRI [12,13,14,15,16,17,18] have all been investigated for evaluating treatment response in GBM. APT-CEST MRI has been shown to be promising in several applications related to imaging human brain tumors, such as differentiating between high- and low-grade tumors, or assessing the effects of treatment on tumor regrowth [19,20,21]. The principal contrast mechanism in APT-CEST is chemical exchange between the amide groups of mobile proteins and the observed brain water signal; APT hyperintensity is therefore usually attributed either to increased protein amide content in actively growing tumors, and/or increased exchange rates, which depend on factors such as pH [22,23]. However, there are also some other possible mechanisms that may lead to contrast in APT-CEST images, depending on how the images are acquired and processed, and on what post hoc corrections are applied. Some of these factors include (a) the effect of changes in brain water T_1_ relaxation times (which are increased in many brain lesions), and (b) significant contrast from the relayed Nuclear Overhauser Effect (NOE) [21]. Therefore, it might be advantageous to have an independent measurement of protein amide levels in the brain.

MRS can also be used to directly observe amide resonances downfield (DF) from water (>4.7 ppm), although care has to be taken to avoid saturation of the water signal, as exchange between water and amides causes attenuation of the amide signals if water suppression using pre-saturation is performed. DF MRS in the human brain has been previously performed using single voxel (SV) spatial localization at various field strengths [11,24,25,26,27], including in a rodent brain tumor model [28]. Recently, techniques for mapping of DF resonances (DF-MRSI, including amides) have been developed and demonstrated for both 2D and 3D encoding at the widely available field strength of 3T [29,30]. While SV localization has a number of advantages for MRS, including optimization of field homogeneity on the target region and short scan times, it does also have a number of limitations, the main one being that it provides little or no information on the spatial distribution of metabolite signals. This is particularly a problem for studies in patients with focal brain lesions, such as tumors, which are notoriously inhomogeneous, and the results obtained will often be highly dependent on the SV MRS voxel placement [31]. SV MRS also becomes inefficient when multiple brain regions are of interest to be examined. In contrast, MRSI allows for spectra from multiple brain regions to be recorded simultaneously and maps of the metabolite resonances to be reconstructed in post-processing [31]. Our recent paper [30] has shown that near whole-brain coverage is possible for DF-MRSI at a nominal spatial resolution of 0.7 cm [3] in a scan time of just over 20 min at the clinically widely available field strength of 3T. This spatial resolution is similar to that of other metabolic imaging techniques in human brain (e.g., fluorodeoxyglucose (FDG)-positron emission tomography (PET), or upfield MRSI) and is sufficient for the study of many different neurological or oncological diseases, including most brain tumors [32].

In addition to the amide resonances of mobile proteins (~8.1 to 8.4 ppm), a number of other compounds have functional groups that may be visible in the downfield region of the spectrum, including the amide resonance of N-acetyl aspartate (NAA, 7.9 ppm), two resonances from the imidazole group of homocarnosine (~7.1 and 8.0 ppm), glutathione (8.3, 8.5 ppm), ATP (~8.2 and 8.5 ppm), and others [24,26]. Some compounds may become more visible in the downfield region under pathological conditions (e.g., phenylalanine, ~7.3–7.4 ppm, in patients with phenylketonuria (PKU) [33]), or with administration of exogeneous substances (e.g., histidine, ~7.1 and 7.8 ppm) [34]. Recently, using a 7T scanner and very large voxel sizes to give high signal-to-noise ratios (SNR), it has also been possible to assign signals to nicotinamide adenine dinucleotide (NAD+, ~8.9, 9.2, 9.3 ppm) and L-tryptophan (L-Trp, ~10.1 ppm) [35]. MRS visibility of DF resonances depends on several factors, including the metabolite concentration and exchange rate with water [29,36]. Even when water suppression using pre-saturation is not used, the linewidths and T_2_ relaxation times of DF resonances will depend on their water exchange rates, and those that are rapidly exchanging with water will not be visible in the DF spectrum. Therefore DF-MRSI is expected to be most sensitive for functional groups that have relatively slow exchange with water, unlike APT-CEST where sensitivity is more optimal for intermediate exchange rates. Ultimately, therefore, DF-MRSI and APT-CEST may be techniques that offer complementary (rather than identical) information of brain amide levels, looking at amide pools that differ in water exchange rates.

This paper reports initial results of 3D DF-MRSI in a small cohort of patients with GBM who were being followed for tumor recurrence post treatment. The purposes of the study were to determine feasibility, as well as to examine possible differences in DF-metabolite levels, focusing on the major 8.18 and 7.90 ppm resonances, between recurrent tumor, normal brain, and treatment effects.

## 2. Methods

### 2.1. Data Acquisition

Seven patients (4F, age range 44–65, mean ± s.d. 59.3 ± 7.5 years) with previously treated GBM were scanned using a recently developed 3D DF-MRSI protocol and a Philips 3T ‘Ingenia Elition’ scanner equipped with a 32-receive channel head coil [37]. This study was approved by the Johns Hopkins University School of Medicine Institutional Review Board and all participants provided written informed consent.

The MR protocol consisted of 3D T_1_-weighted and Fluid Attenuated Inversion Recovery (FLAIR)-weighted images, followed by proton density (PD) localizer images for DF-MRSI.

**Figure 1:**
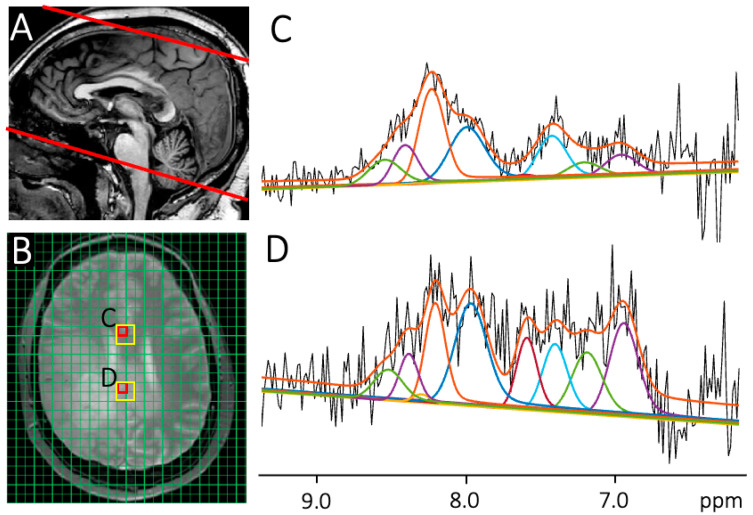
(**A**) Sagittal T_1_-weighted image in patient #7, with red lines indicating the inferior and superior margins of the 12 cm slab covered by DF-MRSI. (**B**) Axial proton density (PD) localizer MRI with 26 × 29 MRSI grid overlaid in green; representative voxel locations chosen for normal-appearing brain and lesion are indicated in red; 4 contiguous voxels were chosen for each region (indicated in yellow). (**C**,**D**) Representative spectra and LCModel fit results for the voxel locations indicated in (**B**). Note that no line-broadening is applied to the spectra in (**C**,**D**).

Three-dimensional DF-MRSI data with a nominal spatial resolution of 7 × 7 × 15 mm were acquired from a field of view of 200 × 180 × 120 mm, matrix size 29 × 26 × 8, scan time 22 m 42 s. A 3D volume consisting of eight 15 mm slices was recorded in oblique axial prescription covering from the base of the cerebellum to the vertex (Figure 1A). Scan parameters were TR 287 ms, TE 22 ms, flip angle 78°, 1 excitation, with an inferior saturation pulse applied. Shimming was performed using the ‘FastMAP’ technique for the optimization of the B_0_ field homogeneity up to 2nd order. Details of the pulse sequence, spectral-spatial excitation, and frequency selective refocusing pulses can be found in reference [30].

A non-water suppressed FID-MRSI scan was also recorded at the same resolution, slice locations, and matrix size as the DF-MRSI. Scan parameters for FID-MRSI were TR 264 ms, TE 1 ms, flip angle 30°, 1 excitation, SENSE acceleration (R = 2), scan time 11 m 14 s.

### 2.2. Diagnosis and Region of Interest Analysis

Longitudinal, clinical MRI scans performed as part of each patient’s routine care, including administration of a contrast agent, were evaluated by a neuroradiologist according to Response Assessment in Neuro-Oncology (RANO) criteria [38], and used to classify lesions observed on the research MR exam as either disease progression or treatment effect. Target regions of interest (ROIs) were identified in each case on the FLAIR images recorded in the same session as DF-MRSI, and these coordinates were then transferred, for instance as shown in Figure 1, for a quantitative analysis of the lesion and uninvolved brain (usually contralateral to the lesion). Usually, 4 MRSI voxels from each region were chosen for analysis (Figure 1B).

### 2.3. DF-MRSI Post-Processing and Quantification

DF data were analyzed using the previously described post-processing pipeline [30]. Briefly, DF signals were frequency corrected on a voxel-by-voxel basis using the H_2_O MRSI data. An HLSVD filter for water removal was then applied before quantification with ‘LCModel’ software [39,40,41,42] using a basis set consisting of 9 Gaussian DF peaks. LCModel usually analyzes in vivo spectra as a linear combination of model spectra from individual metabolites (obtained either via spectral simulation or recoding of in vitro solutions [42,43]); however, in the case of DF MRS, because many of the resonances have not currently been definitively assigned to specific metabolites, DF-MRSI data were analyzed as a linear combination of the 9 individual Gaussian peaks. These peaks were based on prior studies, most notably that of Fichtner et al. [25], who analyzed high-field (9.4T) DF spectra of the human brain and found that the spectra were best characterized by a group of 12 peaks; the 9 values used here reflect the lower field strength (3T) of the current study as well as the smaller frequency range covered using the current pulse sequence (~6.8 to 8.5 ppm); in the 9.4T study, peak frequencies ranged from 5.8 to 8.5 ppm. LCModel performs a least-squares minimization between the model and experimental data, where the model parameters include estimated concentrations, zero and first-order phase corrections, frequency shift, linewidth, and cubic spline baseline correction. The baseline stiffness was set using the control parameter “DKNTMN” = 5. In addition, uncertainty estimates (Cramer-Rao Lower Bounds, CRLB) are provided for each resonance. CRLBs were not used to filter results since this has been suggested to be a potential source of bias [44]; however, for the reported resonances (see below), generally CRLBs were <50% in most brain regions. The control file including all fitting parameters for LCModel is provided in the supplemental information. Examples of LCModel fits in one patient for both lesion and normal-appearing tissue are shown in Figure 1C,D. Concentration values output from the LCModel are reported in ‘institutional units’, since they are not corrected for tissue water content, relaxation times, and other technical factors related to the DF-MRSI acquisition and therefore cannot be equated to conventional biochemical units such as millimolar or micromoles per gram wet weight.

Finally, DF amide (8.18 ppm) and DF7.90 ppm maps were reconstructed from the LCModel peak area estimates and linearly interpolated by a factor of 8 for display purposes. These two resonances were chosen because they are the most prominent signals in the DF spectrum, and therefore give the highest quality metabolic images; in addition, the 8.18 ppm peak is assigned to mobile protein amide groups, which are believed to be increased in brain tumors, and a principal component of the contrast seen in APT-CEST images. Also, the 7.90 ppm peak is often assigned to the amide group of NAA, which is known to be decreased in most brain lesions. Brain masks were calculated from localizer PD MR images and co-aligned with DF maps.

### 2.4. Statistical Analysis

Voxel levels of each metabolite were normalized to the value of the same metabolite in normal-appearing brain voxel (usually in the contralateral hemisphere). After averaging for each lesion, metabolite levels were compared between normal brain, treatment response, and disease progression groups using ‘R 3.5.1’ [45]. General linear regression followed by log transformation was performed to compare brain metabolite levels between ROIs. *p* values were corrected for multiple comparison using the Benjamini–Hochberg method and considered significant if they were less than 0.05 (i.e., statistical significance was set at a false positive rate of 5%). Box and whiskers plots were created using the raw, uncorrected LCModel output for each voxel in ‘institutional’ units.

## 3. Results

Of the seven cases, two were classified as having disease progression, and the other five were classified as treatment effect. The clinical characteristics of the seven patients are given in Table 1. All patients had previously undergone surgical resection, XRT and TMZ, and mean time elapsed since most recent treatment at time of DF-MRSI was 20.4 ± 20.7 weeks (range 4–60 weeks).

**Figure 2:**
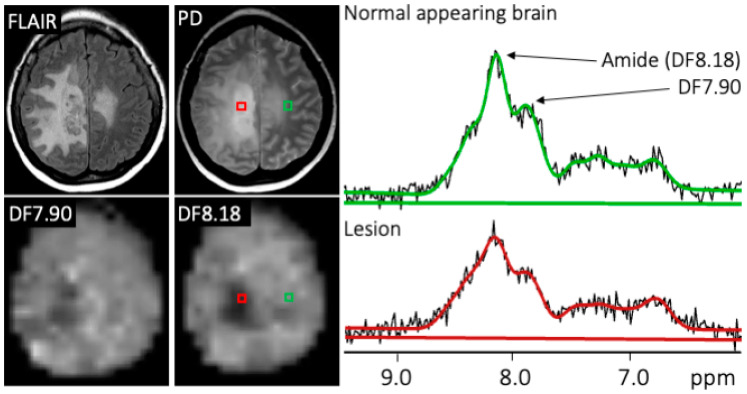
Representative MR images, DF-MRSI maps, and spectra from patient #3, a 45-year-old female with MGMT methylated GBM scanned 14 weeks after adjuvant TMZ, classified as exhibiting treatment response based on clinical improvement and longitudinal MRI scans. DF8.18 and DF7.90 ppm maps and DF spectra show decreased levels of DF8.18 and DF7.90 ppm resonances in the lesion (red) compared to normal-appearing brain (green). Note that spectral signal-to-noise ratios are higher in this figure compared to Figure 1 because an 8 Hz line-broadening has been applied for display purposes.

Figure 2 shows representative MR images, DF-MRSI maps, and selected spectra from one patient classified as treatment effect due to clinical improvement and lack of progression on follow-up standard-of-care MRI. DF8.18 and DF7.90 ppm peak levels are decreased in the lesion compared to the normal-appearing brain.

**Figure 3:**
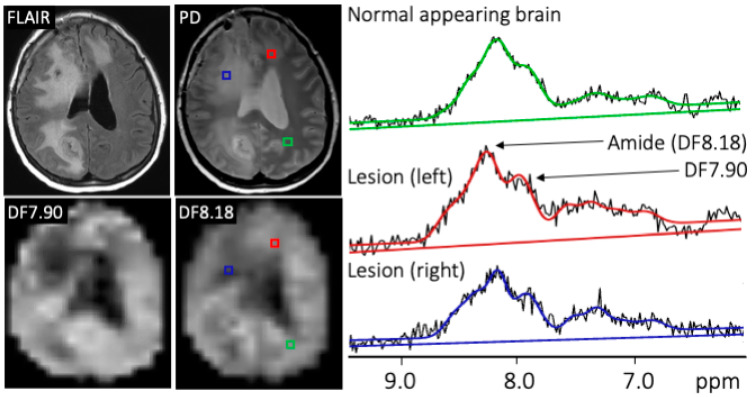
Representative MR images, DF-MRSI maps, and spectra from patient #2, a 63-year-old female with multifocal, MGMT unmethylated GBM, 6 weeks after completing XRT/TMZ. The rapidly progressing left frontal lesion (red voxel) is characterized by slightly increased amplitudes of the DF8.18 and DF7.90 ppm peaks compared to normal-appearing left parietal tissue (green voxel), although visually no hyperintensity is apparent on the DF8.18 maps. The blue voxel in the right hemisphere lesion (a region classified as treatment effect) shows lower levels of both DF8.18 and DF7.90 peaks.

Figure 3 shows representative MR images, DF-MRSI maps, and selected spectra from one patient with multi-focal GBM classified as exhibiting disease progression. DF8.18 levels are slightly increased in the disease progression region compared to the normal-appearing brain.

**Figure 4:**
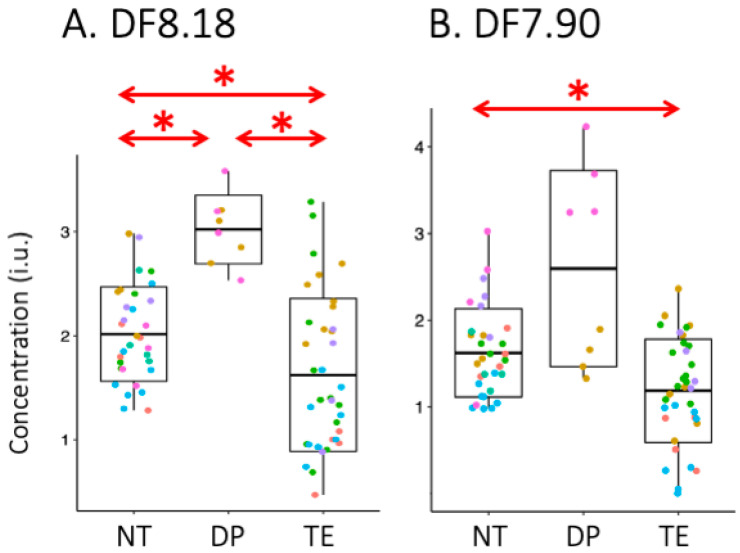
Comparison of (**A**) DF8.18 and (**B**) DF7.90 ppm peak areas from LCModel (‘concentrations in institutional units’, i.u.) between regions classified as normal tissue (NT), disease progression (DP) and treatment effect (TE). Red arrows indicate statistically significant differences between groups (*, *p* < 0.05). It can be seen that DF8.18 levels are higher in DP than both NT and TE; in addition, DF8.18 levels are lower in TE than DP. DF7.90 levels are lower in TE than NT, but other group comparisons were not significant. Box plots show mean, +/− standard deviation and min/max from the whiskers. Color represents individual subjects.

Figure 4 shows non-normalized concentrations of the DF8.18 and DF7.90 peaks for all 76 voxels; normal tissue (NT, *n* = 32), treatment effect (TE, *n* = 36), and disease progression (DP, *n* = 8). At the patient level (*n* = 7), significant differences were found for normalized DF8.18 levels between ROIs classified as disease progression, treatment effect, and normal tissue; *p* = 0.012 for TE vs. DP, *p* = 0.002 for TE vs. NT, *p* = 0.003 for DP vs. NT. For DF7.90 levels, the only significant difference was between ROIs classified as normal tissue and treatment effect (*p* = 0.004) (Table 2).

## 4. Discussion

This study shows that DF-MRSI in human brain tumors at 3T is feasible and was well tolerated by all patients, despite the approximately 20 min scan times. In this preliminary study, DF8.18 resonances were higher in regions classified as disease progression compared to those classified as treatment effect, and also compared to normal brain. The results therefore show promise for monitoring the effects of treatment in patients with GBM, but should also be interpreted with caution, since the sample size is so small, and no reproducibility studies of 3D DF-MRSI have yet been published. Other potential applications (for instance, diagnosis or treatment planning) should also be explored. DF-MRSI has a number of technical advantages compared to traditional upfield proton MRSI, in that lipid suppression is not required, and also shimming (B_0_ field homogeneity) is slightly less critical, due to the somewhat broader natural linewidths of the DF resonances [30].

Although statistically significant, only relatively small elevations of DF8.18 were found in voxels categorized as disease progression compared to normal tissue, and hyperintensity was not visually obvious on DF8.18 images in these cases. This is in contrast to the pronounced hyperintensity commonly observed on APT-CEST, which is often interpreted as due to increased protein amide concentrations. In this regard, DF-MRSI and APT-CEST may provide complementary information, with DF-MRSI measurements being more sensitive for relatively slowly exchanging amide groups, whereas APT-CEST is more sensitive for somewhat faster chemical exchange. Alternatively, the hyperintensity of brain tumors on APT-CEST may be due to factors other than just amide concentration, such as changes in water relaxation times and/or exchange rates [19].

Quantification of metabolite levels in brain tumors is complicated by changes in cellular density and the usually increased water content of the lesions due to blood–brain barrier breakdown, vasogenic edema, and other factors. In many conventional upfield MRSI studies, either the water signal or the total creatine (tCr) signal from the same voxel are used as internal references in order to normalize the signal and estimate concentrations values. In the current study, no attempt was made to use internal referencing for quantification because of the variable water signal and lack of a tCr reference. Instead, lesion levels of each metabolite were normalized to the value of the same metabolite in normal-appearing brain (usually in the contralateral hemisphere). Future work should focus on estimating and correcting for variations in brain and lesion water content to provide a voxel-by-voxel internal reference.

One unexpected result in the current study was similar levels of the DF7.90 peak in ROIs classified as disease progression and normal tissue. In normal brain, this peak is usually assigned as the amide resonance of NAA at ~7.82 ppm [46]. One of the defining, very commonly observed features of upfield MRSI of GBM (and necrosis) is decreased or even absent NAA [47,48]. So, in the current study, it is highly unlikely that the DF7.90 ppm peak measured here in lesions is originating from NAA. It has previously been noted that the DF7.90 ppm peak may have both ‘narrow’ and ‘broad’ components [24,25], with the broad component perhaps arising from amide groups other than NAA; in the current study, it therefore seems likely that those currently unassigned compounds may be responsible for the lack of decreases in the DF7.90 peak seen here in disease progression cases.

It was also observed that normal-appearing brain (NT) showed quite variable levels of the DF8.18 peak amongst the seven volunteers (Figure 4). This spread in NT values probably results from several factors, including possible difference between gray and white matter, the fairly low signal-to-noise ratios in the individual voxels, fitting errors in the LCModel, and also subject to subject variations. Since all patients had prior treatments such as chemoradiation (which can be toxic to normal brain), there may be metabolic changes even in brain regions that do not show obvious abnormalities on MRI.

The study also has a number of limitations, most notably the small sample size of this pilot study, and also the lack of direct pathological confirmation. Therefore, the initial results reported here will require confirmation in larger numbers of subjects, ideally with pathologically confirmed diagnoses. We also expect that with further technical development, DF-MRSI will be possible with somewhat higher spatial resolution (e.g., more, thinner slices) and also reduced scan time, for instance by using sparse-sampling and low-rank reconstruction methods [37]. Additionally, it is hoped that with further work it will be possible to improve quantitation and assignment of specific peaks in the downfield spectrum, so that compounds other than amide resonances (and the DF7.90 peak) may be investigated, to improve the understanding of biochemical changes in human brain tumors. Finally, future studies may include concurrent APT-CEST imaging to allow for direct comparison of the diagnostic value of APT-CEST and DF-MRSI in patients with brain tumors, as well as comparison to other advanced MR techniques that are commonly used to evaluate treatment response (such as DWI, PWI, and upfield MRSI).

## Figures and Tables

**Table 1:** Clinical characteristics of patient population. All tumors were GBM (WHO grade IV, IDH wild type). MGMT = O^6^-methylguanine-DNA methyltransferase, TERT = telomerase reverse transcriptase, EGFR = epidermal growth factor receptor, CDKN2A = cyclin dependent kinase inhibitor 2A, PIK3CA = phosphatidylinositol-4,5-bisphosphate 3-kinase subunit alpha, NF1 = neurofibromatosis type 1, NGS = next generation sequencing. F = female, M = male, XRT= external beam radiotherapy, TMZ = temozolomide.

Case	M/F	Age	Molecular Diagnosis	Treatment History	Time Since End of Last XRT/TMZ Treatment
**1**	F	59	MGMT unmethylated	Surgery × 5, XRT/TMZ × 2, adjuvant TMZ × 10 cycles	60 weeks (2nd course)
**2**	F	63	MGMT unmethylated, TERT, EGFR, CDKN2A deletion	Surgery, XRT/TMZ	6 weeks
**3**	F	45	MGMT methylated, TERT, NF1, CDKN2A deletion	Surgery, XRT/TMZ, adjuvant TMZ × 1 cycle	14 weeks (adjuvant TMZ: 4 weeks)
**4**	F	64	MGMT methylation, TERT, EGFR, PIK3CA	Surgery with Gliadel, XRT/TMZ × 2, adjuvant TMZ × 6 cycles, Optune device	4 weeks (2nd course)
**5**	M	66	MGMT unmethylated. No NGS/molecular testing	Surgery, XRT/TMZ, adjuvant TMZ × 5 cycles	35 weeks
**6**	M	59	MGMT unmethylated, TERT, NF1	Surgery, XRT/TMZ, adjuvant TMZ × 5 cycles	20 weeks (adjuvant TMZ: 2 weeks)
**7**	M	66	MGMT unmethylated, TERT, EGFR amplification, CDKN2A deletion	Surgery, XRT/TMZ	4 weeks

**Table 2:** Results of the statistical analysis at the patient level (*n* = 7). [DF8.18] = concentration value of 8.18 ppm peak and [DF7.90] concentration of DF7.90 ppm peaks. The ‘n’ subscript indicates concentration values normalized to normal appearing brain ‘control’ region. TE = treatment response, NT = normal tissue, DP = disease progression.

	NT Mean ± s.d.	DP Mean ± s.d.	TE Mean ± s.d.	TE vs. DP (*p*-Value)	TE vs. NT (*p*-Value)	DP vs. NT (*p*-Value)
[Amide]_n_	1.00 ± 0.00	1.5 ± 0.4	0.7 ± 0.2	0.012	0.002	0.003
[DF7.90]_n_	1.00 ± 0.00	1.4 ± 0.7	0.7 ± 0.2	0.058	0.004	0.083

## Data Availability

Data is available on request.

## Supplementary Materials

The following supporting information can be downloaded at: https://www.mdpi.com/article/10.3390/cancers15174311/s1, The control file including all fitting parameters for LCModel is provided in the supplemental information.
